# Prognostic Impact of the UMIPIC Program in the Follow Up in Patients with Heart Failure and Cardiorenal Syndrome

**DOI:** 10.3390/jcm12237261

**Published:** 2023-11-23

**Authors:** Manuel Méndez-Bailón, Noel Lorenzo-Villalba, Álvaro González-Franco, Luis Manzano, Jesús Casado-Cerrada, José M. Cerqueiro, José Pérez-Silvestre, José Carlos Arévalo-Lorido, Alicia Conde-Martel, Melitón Francisco Dávila-Ramos, Margarita Carrera-Izquierdo, Emmanuel Andrès, Manuel Montero-Pérez-Barquero

**Affiliations:** 1 Internal Medicine Department, Hospital Clínico San Carlos, Instituto de Investigación Sanitaria del Hospital Clinico San Carlos (IdISSC), Universidad Complutense, 28040 Madrid, Spain; 2 Internal Medicine Department, Hôpitaux Universitaires de Strasbourg, 67000 Strasbourg, France; 3 Internal Medicine Department, Hospital Universitario Central de Asturias, 33011 Oviedo, Spain; alvarogfranco@yahoo.com; 4 Internal Medicine Department, Hospital Universitario Ramón y Cajal, Instituto Ramon y Cajal de Investigación Sanitaria (IRYCIS), Universidad de Alcala de Henares, 28034 Madrid, Spain; 5 Internal Medicine Department, Hospital Universitario de Getafe, 28905 Madrid, Spain; 6 Internal Medicine Department, Hospital Universitario Lucus Augusti, 27003 Lugo, Spain; 7 Internal Medicine Department, Consorcio Hospital General Universitario de Valencia, 46014 Valencia, Spain; jopesilver@hotmail.com; 8 Internal Medicine Department, Hospital Universitario de Badajoz, 06080 Badajoz, Spain; joscarlor@gmail.com; 9 Internal Medicine Department, Hospital Universitario de Gran Canaria Dr. Negrín, 35010 Gran Canaria, Spain; 10 Internal Medicine Department, Hospital Universitario Nuestra Señora de la Candelaria, 38010 Tenerife, Spain; 11 Internal Medicine Department, Complejo Hospitalario de Soria, 42005 Soria, Spain; 12 Internal Medicine Department Hospital Universitario Reina Sofía, Instituto de Investigación Biomédica de Cordoba (IMIBIC), 14004 Cordoba, Spain; montero.manolo@gmail.com

**Keywords:** heart failure, cardiorenal syndrome, UMIPIC

## Abstract

Background: Individuals suffering from heart failure (HF) and cardiorenal syndrome (CRS) represent a special group of patients considering their age, multiple health issues, and treatment challenges. These factors make them more susceptible to frequent hospital stays and a higher mortality rate. UMIPIC is a multidisciplinary care model program for patients with heart failure follow up provided by internists and nurses who are experts in this entity. Our study delved into the effectiveness of this specialized care program (UMIPIC) in mitigating these risks for HF and CRS patients. Methods: We analyzed the medical records of 3255 patients diagnosed with HF and CRS types 2 and 4, sourced from the RICA registry. These patients were divided into two distinct groups: those enrolled in the UMIPIC program (1205 patients) and those under standard care (2050 patients). Using propensity score matching, we ensured that both groups were comparable. The study focused on tracking hospital admissions and mortality rates for one year after an HF-related hospital stay. Results: Patients in the UMIPIC group experienced fewer hospital readmissions due to HF compared to their counterparts (20% vs. 32%; Hazard Ratio [HR] = 0.48; 95% Confidence Interval [95% CI]: 0.40–0.57; *p* < 0.001). They also showed a lower mortality rate (24% vs. 36%; HR = 0.64; 95% CI: 0.54–0.75; *p* < 0.001). Furthermore, the UMIPIC group had fewer total hospital admissions (36% vs. 47%; HR = 0.58; 95% CI: 0.51–0.66; *p* < 0.001). Conclusions: The UMIPIC program, centered on holistic and ongoing care, effectively reduces both hospital admissions and mortality rates for HF and CRS patients after a one-year follow-up period.

## 1. Introduction

Heart failure (HF) constitutes the principal etiological factor for hospital admissions among individuals aged 65 and above, contributing to 3% of all hospitalizations and accounting for 2.5% of healthcare expenditure in Spain [1,2]. A contemporaneous investigation conducted in 2019 identified a prevalence of 1.89% with an incidence rate of 2.78 new cases per 1000 person-years within the Spanish population [3].

Renal dysfunction emerges as a prominent comorbidity in patients enduring chronic heart failure and becomes particularly accentuated during episodes of acute heart failure (AHF) [4,5]. In the milieu of AHF, a deterioration in renal function is observed in a substantial 10–40% of the patient cohort [6,7]. Despite its significant prevalence, this decline in renal function remains an intricate obstacle in terms of accurate diagnosis, prognostication, and therapeutic intervention [6]. Cardiorenal syndrome (CRS), defined as the concurrent dysfunction of both cardiac and renal systems, represents a clinical and therapeutic challenge. The reciprocal relationship between cardiac and renal dysfunction in CRS has been discussed in a recent consensus classification [6].

The Heart Failure and Atrial Fibrillation Working Group, an affiliate of the Spanish Society of Internal Medicine (SEMI), has pioneered a specialized care initiative named UMIPIC (Comprehensive Management Units for Patients with HF). The efficacy of UMIPIC in attenuating hospital admissions and mortality among the general HF patient population has been well-documented [8]. However, the impact of this interventional framework on patients co-diagnosed with HF and CRS remains an unexplored domain.

The present study is designed to rigorously evaluate the efficacy of the UMIPIC program in diminishing hospitalization rates and overall mortality over a one-year follow-up period, specifically for patients with coexistent HF and CRS.

## 2. Methods

We conducted a prospective observational study of two cohorts of patients who were followed from hospital discharge in a non-randomized manner with a one-year follow-up. Patients enrolled in the RICA registry from March 2008 to March 2020 and diagnosed with heart failure (HF) and cardiorenal syndrome (CRS) were eligible for inclusion. Specifically, the subset of patients exhibiting renal dysfunction—defined by a glomerular filtration rate below 60 mL/min/m^2^ for a duration of no less than three months—was extracted from the comprehensive registry for further study. According to Ronco et al.’s [9] classification, this study included patients with Type 2 CRS and Type 4 CRS. The remaining subtypes have not been included in this study since we have not included patients with CRS (types 1 and 3), or patients with systemic diseases that trigger secondary heart failure such as sepsis. 

The RICA registry is a multi-institutional, prospective cohort database that incorporates patients aged 50 and above who are consecutively admitted due to episodes of decompensated HF, in line with the diagnostic criteria set forth by the European Society of Cardiology.

Data were collected in an anonymized manner through a dedicated web portal (https://www.registrorica.org, accessed on 18 October 2023). The coordination of data collection is overseen by the Heart Failure and Atrial Fibrillation Working Group, a constituent of the Spanish Society of Internal Medicine (SEMI). Ethical approval for the study was granted by the Clinical Research Ethics Committee of Hospital Universitario Reina Sofía de Córdoba, and informed consent was secured from all participating patients prior to their inclusion in the study.

Patients were excluded from the study if they required specialized cardiological interventions such as ischemic procedures requiring catheterization, device implantation, or valve prostheses, or if they were in advanced functional states pending cardiac transplantation. Additionally, patients with functional and cognitive limitations who lacked sufficient social and familial support were also considered ineligible for inclusion in the study.

### 2.1. Overview of the UMIPIC Program

The UMIPIC initiative provides a holistic, ongoing care model for elderly patients grappling with complex, chronic heart failure (HF). Exclusion criteria include the need for specialized cardiology services such as catheterization, device implantation, and valve replacements, and those awaiting heart transplantation. Additionally, insufficient socio-familial support for patients with cognitive and functional impairments warrants exclusion.

UMIPIC units are staffed by qualified nurses and operate under a stringent, protocol-driven program focused on five core pillars: (1) inclusive clinical oversight covering HF and associated comorbidities; (2) continuous patient monitoring through in-person visits, telephonic consultations, and in-hospital care; (3) structured patient and caregiver education on self-care and support; (4) prompt accessibility for non-scheduled decompensation episodes; (5) direct coordination with specialty physicians like cardiologists and nephrologists. The program includes consultations from both medical and nursing professionals.

### 2.2. Selection Criteria and Statistical Methods

For this study, patients were included based on the presence of renal dysfunction, indicated by a glomerular filtration rate below 60 mL/min/m^2^ persisting for at least three months. Upon discharge, patients were categorized into either the UMIPIC cohort or the conventionally treated group. Baseline data encompassed sociodemographic factors, clinical history, analytical and imaging parameters, and therapeutic interventions.

Key metrics for evaluating outcomes were total hospitalizations at one year, HF-specific admissions during the initial month and at one year, and all-cause mortality at the one-year mark. Quantitative variables were summarized as median values [interquartile range], and Student’s *t*-tests or Wilcoxon tests were employed depending on sample distribution normality. Categorical variables were reported as frequencies and percentages, and compared using Chi-square tests with Yates’ correction as needed, or Fisher’s exact tests.

To minimize selection bias, propensity score matching was performed via Cox regression. This included variables with significant differences between the UMIPIC and non-UMIPIC cohorts, except treatment-related variables. Matching was executed on a 1:1 basis without replacement and with a caliper width of 0.2 standard deviations for the propensity score, utilizing nearest-neighbor matching.

Survival analyses were carried out through Kaplan–Meier estimators, and differences between survival curves were assessed using log-rank tests. A *p*-value less than 0.05 was considered statistically significant. Statistical computations were executed on SPSS version 28 and the MatchIt R 3.2 package.

### 2.3. Ethical Compliance

The study secured approval from the ethics committee at the University Hospital of Cordoba and adheres to the ethical tenets laid down in the Declaration of Helsinki.

## 3. Results

**Figure 1:**
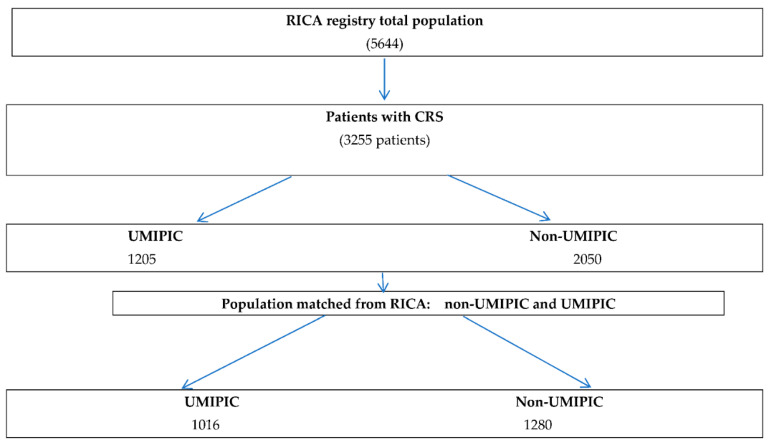
Patient flow chart. Legend: RICA registry (Registro Nacional de Pacientes con Insuficiencia Cardiaca) (RICA); CRS: Cardiorenal syndrome; Unidad de Manejo Integral del Paciente con Insuficiencia Cardiaca (UMIPIC).

From March 2008 through March 2020, a total of 3255 patients were enrolled in the study, comprising 1205 individuals in the UMIPIC cohort and 2050 in the non-UMIPIC cohort (as depicted in Figure 1). The median age across all participants was 82 years, with women constituting 56% of the study population. Comprehensive demographic and clinical attributes for each group are detailed in Table 1. Notably, participants in the UMIPIC cohort were of a slightly higher average age (83 vs. 81; *p* < 0.01) and exhibited a greater prevalence of comorbid conditions as per the Charlson Comorbidity Index (3.79 vs. 3.54; *p* = 0.007). Conversely, cognitive function was comparatively diminished in the RICA cohort, as evidenced by the higher scores on the Pfeiffer Index (1.86 vs. 1.49) (refer to Table 1).

**Figure 2:**
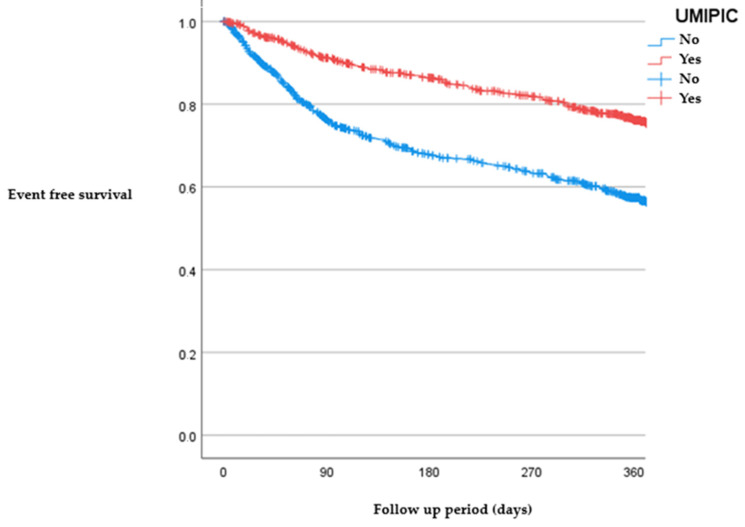
Kaplan–Meier curves for HF admission during the follow-up year.

**Figure 3:**
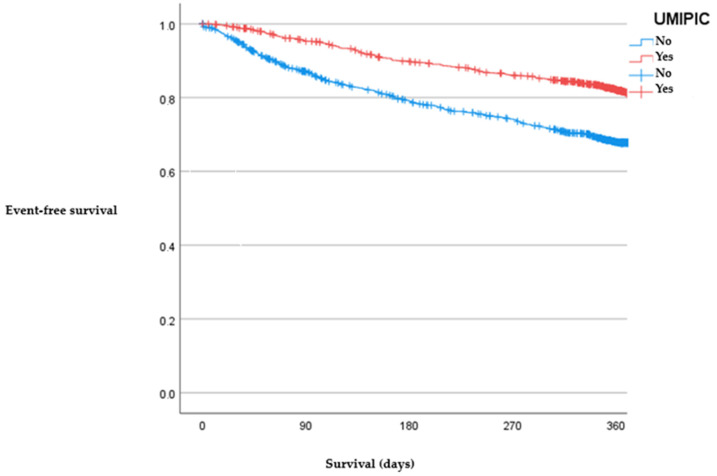
Kaplan–Meier curves for mortality during the follow-up year.

In order to mitigate the disparities in baseline characteristics between the two cohorts (as outlined in Table 2), we employed a propensity score matching analysis via logistic regression. This analysis included all variables that exhibited a statistically significant divergence between the non-UMIPIC and UMIPIC groups, with the exception of those specifically related to treatment protocols. The nearest-neighbor matching technique was utilized for this purpose. Survival outcomes were subsequently evaluated through Kaplan–Meier survival curves, and a comparative analysis of these curves was conducted using the log-rank test (as illustrated in Figure 2 and Figure 3).

Table 3 shows the admissions and overall mortality in both groups (UMIPIC vs. non-UMIPIC) during the 12 months of follow-up. As shown in this table, patients followed up in the UMIPIC program had fewer admissions for both heart failure (HR 0.48 (0.39–01)) and other causes (HR 0.56 (0.47–0.68)) and lower mortality (HR 0.55 (0.45–0.67)).

Figure 2 and Figure 3 show a benefit for both the survival (Long Rank: 79.5 (*p* < 0.001); HR (UMIPIC group) = 0.48 (0.40–0.57)) and mortality of patients (Long Rank: 31.5 (*p* < 0.001); HR (UMIPIC group) = 0.64 (0.54–0.75)) followed in the UMIPIC program.

## 4. Discussion

This research highlights that the integration of the UMIPIC care model in managing patients with heart failure (HF) and cardiorenal syndrome (CRS) led to significant reductions in both hospital admissions and mortality rates over a one-year observation period. Earlier studies have corroborated the efficacy of the UMIPIC program in a broader HF patient population; however, our study specifically focuses on the subgroup afflicted with both HF and CRS, an area yet to be explored in prior research [10]. We believe it is important to highlight that UMIPIC demonstrated clinical benefits in patients with HF and cardiorenal syndrome. More than 50% of our patients with HF who are admitted to our hospitals have associated renal insufficiency. This comorbidity is associated with a higher number of complications and readmissions in HF patients. Patients with cardiorenal syndrome more frequently present refractory pulmonary congestion with the need for additional diuretics. In addition, chronic renal insufficiency associated with HF limits the use of treatments that modify the evolution of the disease, such as ACE inhibitors, ARABs, and antialdosteronics [11,12,13,14]. Despite these limitations, our study shows that these patients benefit from follow-up in UMIPIC.

Our study sample significantly diverges from those commonly observed in clinical HF trials [15,16]; notably, our subjects are a decade older, primarily female, and present with multiple comorbidities, classifying them as high-risk individuals. Therefore, the comprehensive and tailored UMIPIC program proves especially advantageous for managing these patients with elevated risk factors and higher rates of hospitalization and mortality [10,17,18]. In this study, both cohorts, those followed up both in UMIPIC and non-UMIPIC, were followed up after discharge in both follow-up arms by internists who participate in the RICA registry and who have knowledge of and interest in the field of heart failure. Hence, a low event rate was observed. Even so, the multidisciplinary follow-up arm through the UMIPIC program has shown more benefit in hospitalization and readmissions.

We found a prompt divergence in the Kaplan–Meier curves, indicating that benefits in reducing HF-related hospitalizations manifest early, likely due to effective “transition to discharge” strategies implemented in the UMIPIC program. Additionally, we found that patients with HF and CRS are more frequently hospitalized for non-HF-related issues. This further emphasizes the need for a multidisciplinary approach that can address the complex medical needs of these patients, a role well-served by internal medicine specialists and nursing staff alike. Furthermore, the UMIPIC program demonstrated an ability to lower not just hospital admissions but also mortality rates [8], a result not consistently observed in other care programs [19]. Our study supports this care model, showcasing its ability to impact both metrics positively. In fact, the efficacy of the UMIPIC model could be potentially understated given the more proactive follow-up protocols in the control population (the RICA group) when compared to other observational studies [20]. Given these findings, we advocate for the referral of HF and CRS patients to units that offer proven, effective care, such as the UMIPIC program.

This study has limitations. As an observational study, eliminating all biases was not feasible, although we mitigated this through propensity score matching. In this sense, since it is not a randomized study, we can only speak of an association in terms of clinical benefit. Furthermore, our data set did not include emergency department visits, which could potentially further underscore the UMIPIC program’s efficacy. Moreover, due to the age and comorbidity profile of our sample, the findings may not be universally applicable, especially to cohorts managed within cardiology or other specialties. Lastly, the exact reasons for hospitalizations unrelated to HF decompensation remain unclear, but likely involve factors outside the scope of the UMIPIC program, necessitating further exploration.

## 5. Conclusions

In patients struggling with heart failure (HF) and cardiorenal syndrome (CRS), the UMIPIC program markedly lowers the likelihood of both hospital readmissions and mortality within the first year post-discharge for HF. While these initial findings are promising, additional research is essential to validate the results, explore the program’s long-term benefits, and delve deeper into the reasons for hospitalizations unrelated to heart failure.

## Figures and Tables

**Table 1:** Basal characteristics of heart failure patients from RICA: total non-UMIPIC and UMIPIC.

Variable	Total (*n* = 3255)	Non-UMIPIC (*n* = 2050)	UMIPIC (*n* = 1205)	*p*
Demographic data				
Age, years (median, range)	82 (77–86)	81 (76–86)	83 (79–88)	<0.01
Sex, women (%)	1850 (56.8)	1146 (55.9)	704 (58.4)	0.164
Comorbidities				
Hypertension (*n*,%)	2932 (90.1)	1827 (89.1)	1105 (91.7)	0.018
Diabetes mellitus (*n*,%)	1631 (50.1)	1044 (50.9)	587 (48.7)	0.231
Dyslipidemia ^a^ (*n*,%)	1776 (54.6)	1099 (53.6)	677 (56.2)	0.155
Ischemic cardiopathy (*n*,%)	871 (26.8)	587 (28.6)	284 (23.6)	0.002
Atrial fibrillation (*n*,%)	1702 (52.3)	1049 (51.2)	653 (54.2)	0.102
COPD (*n*,%)	701 (21.5)	454 (22.1)	247 (20.5)	0.289
Cancer (*n*,%)	393 (12.1)	219 (10.7)	174 (14.4)	0.002
Anemia ^b^ (*n*,%)	325 (10.0)	156 (7.6)	169 (14.0)	<0.001
Peripheral vascular disease (*n*,%)	398 (12.2)	252 (12.3)	146 (12.1)	0.912
Stroke (*n*,%)	487 (15.0)	297 (14.5)	190 (15.8)	0.334
Liver disease ^c^ (*n*,%)	66 (2.0)	45 (0.2)	21 (1.7)	0.440
Dementia (*n*,%)	177 (5.4)	118 (5.8)	59 (4.9)	0.337
Nursing home resident (*n*,%)	258 (7.9)	196 (9.6)	62 (5.1)	<0.001
Charlson index (mean ± SD)	3.63 ± 2.63	3.54 ± 2.56	3.79 ± 2.59	0.007
Clinical characteristics				
NYHA (mean ± SD)	2.39 ± 0.65	2.38 ± 0.67	2.42 ± 0.61	0.058
Barthel index (mean ± SD)	79.9 ± 23.4	80.5 ± 23.5	79.1 ± 23.2	0.092
Pfeiffer index (mean ± SD)	1.72 ± 2.1	1.86 ± 2.2	1.49 ± 1.8	<0.001
SBP, mmHg (mean ± SD)	136.3 ± 26.1	137.6 ± 26.9	134.2 ± 24.4	<0.001
DBP, mmHg (media ± SD)	73.3 ± 15.2	74.5 ± 15.4	71.2 ± 14.5	0.001
Heart rate (mean ± SD)	84.2 ± 21.6	85.6 ± 22.4	81.9 ± 20.0	0.001
BMI (mean ± SD)	29.2 ± 8.6	29.1 ± 8.8	29.3 ± 8.2	0.479
LVEF (%) (mean ± SD)	51.6 ± 15.5	51.0 ± 15.6	52.6 ± 15.2	0.004
Laboratory				
Hemoglobin, g/dL (mean ± SD)	11.7 ± 2.0	11.7 ± 2.0	11.6 ± 1.9	0.058
Creatinine, mg/dL (mean ± SD)	1.7 ± 2.2	1.7 ± 0.7	1.7 ± 3.5	0.603
Glomerular filtration rate MDRD, mL min/1.73 m^2^, (mean ± SD)	41.1 ± 12.1	40.9 ± 12.4	41.4 ± 11.6	0.331
Sodium, mEq/dL (mean ± SD)	138.6 ± 5.8	138.7 ± 5.8	138.5 ± 5.7	0.221
Potassium, mEq/dL (mean ± SD)	4.4 ± 0.6	4.4 ± 0.6	4.4 ± 0.6	0.529
NT-proBNP (mean ± SD)	8395 ± 15,614	8182 ± 17,598	8858 ± 10,041	0.434
Treatment				
Loop diuretics (*n*,%)	2451 (75.3)	1529 (74.6)	922 (76.5)	0.223
ACEI or ARB (*n*,%)	1980 (60.8)	1291 (63.0)	689 (57.2)	0.001
Mineralocorticoids (*n*,%)	683 (21.0)	402 (19.6)	281 (23.3)	0.013
Beta blockers (*n*,%)	2248 (69.1)	1394 (68.0)	854 (70.9)	0.091
Statins (*n*,%)	1030 (31.6)	629 (30.7)	401 (33.3)	0.128
Vitamin K antagonists (*n*,%)	1031 (31.7)	673 (32.8)	358 (29.7)	0.067
Antiaggregant (*n*,%)	968 (29.7)	630 (30.7)	338 (28.0)	0.112
Insulin (*n*,%)	789 (24.2)	510 (24.9)	279 (23.2)	0.271
Biguanide (*n*,%)	457 (14.0)	297 (14.5)	160 (13.3)	0.347

**Legend:** COPD: chronic obstructive pulmonary disease; NYHA: New York Heart Association; SBP: systolic blood pressure; DBP: diastolic blood pressure, BMI: body mass index; LVEF: left ventricular ejection fraction; NT-proBNP: N-terminal prohormone of brain natriuretic peptide; ACEI: angiotensin-converting enzyme inhibitor; ARB: angiotensin II receptor antagonist. ^a^ Dyslipidemia: total cholesterol < 190 mg/dL, LDLc > 115 mg/dL. ^b^ Anemia: hemoglobin < 13 mg/dL in men, <12 gr/dL in women. ^c^ Liver disease: elevated AST or ALT > 3 times the upper limit of normal.

**Table 2:** Baseline characteristics of the matched population from RICA: total, non-UMIPIC, and UMIPIC.

Variable	Total (*n* = 2296)	Non-UMIPIC (*n* = 1280)	UMIPIC (*n* = 1016)	*p*
Demographic data				
Age, years (median, range)	83 (75–91)	82 (74–90)	84 (76–92)	0.010
Sex, women (%)	1331 (58.0)	725 (56.6)	606 (59.6)	0.148
Medical antecedents				
Hypertension (*n*,%)	2113 (92.0)	1178 (92.0)	935 (92.0)	0.529
Diabetes mellitus (*n*,%)	1115 (48.6)	633 (49.5)	482 (47.4)	0.355
Dyslipidemia ^a^ (*n*,%)	1256 (54.7)	697 (54.5)	559 (55.0)	0.800
Ischemic cardiopathy (*n*,%)	557 (24.3)	333 (26.0)	224 (22.0)	0.030
Atrial fibrillation (*n*,%)	1244 (54.2)	687 (53.7)	557 (54.8)	0.584
COPD (*n*,%)	496 (21.6)	294 (23.0)	202 (19.9)	0.083
Cancer (*n*,%)	323 (14.1)	169 (13.2)	154 (15.2)	0.184
Anemia ^b^ (*n*,%)	233 (19.1)	96 (17.4)	137 (20.4)	0.188
Peripheral vascular disease (*n*,%)	258 (11.2)	146 (11.4)	112 (11.0)	0.790
Stroke (*n*,%)	319 (13.9)	168 (13.1)	151 (14.9)	0.249
Liver disease ^c^ (*n*,%)	36 (1.6)	20 (1.6)	16 (1.6)	1.000
Dementia(*n*,%)	113 (4.9)	62 (4.8)	51 (5.0)	0.847
Nursing home resident (*n*,%)	99 (4.3)	59 (4.6)	40 (3.9)	0.470
Charlson index (mean ± SD)	3.59 ± 2.6	3.49 ± 2.5	3.73 ± 2.6	0.028
Clinical characteristics				
NYHA (mean ± SD)	2.42 ± 0.6	2.40 ± 0.6	2.44 ± 0.6	0.174
Barthel index (mean ± SD)	81.6 ± 21.4	83.2 ±19.9	79.5 ± 22.9	0.015
Pfeiffer index (mean ± SD)	1.5 ± 1.8	1.5 ± 1.8	1.4 ± 1.7	0.061
SBP, mmHg (mean ± SD)	134.9 ± 25.3	135.9 ± 25.9	133.7 ± 24.4	0.038
DBP, mmHg (media ± SD)	71.4 ± 14.0	72.2 ± 14.2	70.5 ± 13.8	0.044
Heart rate (mean ± SD)	81.9 ± 19.8	82.8 ± 20.3	80.8 ± 19.2	0.013
BMI (mean ± SD)	28.9 ± 7.0	28.7 ± 5.5	29.1 ± 8.5	0.158
LVEF (%) (mean ± SD)	52.5 ± 15.0	52.1 ± 14.9	53.0 ± 15.2	0.157
Laboratory				
Hemoglobin, g/dL (mean ± SD)	11.6 ± 1.9	11.6 ± 2.0	11.6 ± 1.9	0.339
Creatinine, mg/dL (mean ± SD)	1.7 ± 2.6	1.6 ± 0.6	1.7 ± 3.8	0.547
Glomerular filtration rate MDRD, mL min/1.73 m^2^, (mean ± SD)	41.1 ± 11.9	40.8 ± 12.2	41.4 ± 11.6	0.236
Sodium, mEq/dL (mean ± SD)	138.6 ± 5.6	138.7 ± 6.2	138.4 ± 4.7	0.173
Potassium, mEq/dL (mean ± SD)	4.4 ± 0.6	4.4 ± 0.7	4.5 ± 0.6	0.469
NT-proBNP (mean ± SD)	8629 ± 17120	8392 ± 19752	9077 ± 10474	0.524
Treatment				
Loop diuretics (*n*,%)	1750 (76.0)	973 (76.5)	777 (76.5)	0.805
ACEI or ARB (*n*,%)	1333 (58.1)	759 (59.3)	574 (56.5)	0.187
Mineralocorticoids (*n*,%)	530 (23.1)	273 (21.3)	257 (25.3)	0.028
Beta blockers (*n*,%)	1579 (68.8)	861 (67.3)	718 (70.7)	0.085
Statins (*n*,%)	724 (31.5)	393 (30.7)	331 (32.6)	0.343
Vitamin K antagonists (*n*,%)	755 (32.9)	443 (34.6)	312 (41.3)	0.049
Antiaggregants (*n*,%)	648 (28.2)	365 (28.5)	283 (27.9)	0.744
Insulin (*n*,%)	529 (23.0)	299 (23.4)	230 (22.6)	0.690
Biguanide (*n*,%)	305 (13.3)	181 (14.1)	124 (12.2)	0.194

**Legend:** COPD: chronic obstructive pulmonary disease; NYHA: New York Heart Association; SBP: systolic blood pressure; DBP: diastolic blood pressure, BMI: body mass index; LVEF: left ventricular ejection fraction; NT-proBNP: N-terminal prohormone of brain natriuretic peptide; ACEI: angiotensin-converting enzyme inhibitor; ARB: angiotensin II receptor antagonist. ^a^ Dyslipidemia: total cholesterol < 190 mg/dl, LDLc >115 mg/dl. ^b^ Anemia: hemoglobin < 13 mg/dl in men, <12 gr/dl in women. ^c^ Liver disease: elevated AST or ALT > 3 times the upper limit of normal.

**Table 3:** Mortality and admissions data in the matched population from RICA: total, non-UMIPIC, and UMIPIC.

Matched Population					
Patients	Total (*n* = 2220)	Non-UMIPIC (*n* = 1227)	UMIPIC (*n* = 993)	*p*	HR (95% CI)
Admission for HF at 12 months, *n* (%) *	599 (26.9)	396 (32.3)	203 (20.4)	<0.001	0.48 (0.40–0.57)
Total admissions at 12 months, *n* (%) *	934 (42.1)	572 (46.6)	362 (36.4)	<0.001	0.58 (0.51–0.66)
Mortality at 12 months, *n* (%) *	680 (30.6)	438 (35.6)	242 (24.4)	<0.001	0.64 (0.54–0.75)

**Legend:** * number of admissions and % for 100 patients and year follow up.

## Data Availability

The data presented in this study are available on request from the corresponding author. The data are not publicly available due to privacy restrictions.
